# The association of periodontal disease and oral health with hypertension, NHANES 2009–2018

**DOI:** 10.1186/s12889-023-16012-z

**Published:** 2023-06-12

**Authors:** Yuting Li, Xiaojing Yuan, Qiutong Zheng, Fengxin Mo, Shiheng Zhu, Tianran Shen, Wenhan Yang, Qingsong Chen

**Affiliations:** 1 School of Public Health, Guangdong Pharmaceutical University, Guangzhou, 510006 Guangdong Province China; 2 Guangdong Provincial Engineering Research Center of Public Health Detection and Assessment(2019GCZX012), Guangdong Pharmaceutical University, Guangzhou, 510310 Guangdong Province China

**Keywords:** Oral health, Periodontal disease, Smoking, Hypertension

## Abstract

**Background:**

Hypertension is a worldwide public health problem. We sought to explore the interaction of oral health and smoking on hypertension, and periodontal disease and smoking on hypertension.

**Methods:**

We included 21,800 participants aged ≧ 30 years from the National Health and Nutrition Examination Survey (NHANES) 2009–2018. Information of oral health and periodontal disease were self-reported. Blood pressure was taken by trained personnel and/or physicians at mobile testing center. Multiple logistic regression was used to estimate the association between oral health, periodontal disease and the prevalence of hypertension. The effects of oral health and periodontal disease on hypertension under smoking status and age were analyzed by stratified and interaction analysis.

**Results:**

A total of 21,800 participants were investigated, including 11,017 (50.54%) in hypertensive group and 10,783 (49.46%) in non-hypertensive group. Compared with the excellent/very good of oral health, the multivariable-adjusted OR of good, fair, and poor were 1.13 (95% CI, 1.02–1.27), 1.30 (95% CI, 1.15–1.47), and 1.48 (95% CI, 1.22–1.79) (*p* for trend < 0.001) for hypertension, respectively. Compared without periodontal disease group, the multivariable-adjusted OR of periodontal disease for hypertension was 1.21 (95% CI ,1.09–1.35) (*p* for trend < 0.001). Furthermore, we found the interactions between periodontal disease and smoking, oral health and smoking, periodontal disease and age, oral health and age were *p* < 0.001.

**Conclusions:**

An association between oral health and periodontal disease with the prevalence of hypertension was identified. There exists interactive effect of periodontal disease and smoking, oral health and smoking, periodontal disease and age, oral health and age on hypertension in American population over 30 years of age and older.

**Supplementary Information:**

The online version contains supplementary material available at 10.1186/s12889-023-16012-z.

## Introduction

Hypertension is a common chronic noncommunicable disease that affects the health of people worldwide and causes 10.4 million deaths each year [1]. About a third of the world’s population is reported to be affected by high blood pressure, and premature deaths due to it have risen 56.1% in the past decade [2]. According to 2017 American College of Cardiology/American Heart Association guidelines, the overall crude prevalence of hypertension in American adults was 45.6% [3]. Hypertension is not only a high morbidity and mortality rate, but also a serious consumption of medical and social resources and a heavy economic burden on families and society [4].

Unhealth oral conditions and periodontal disease are important factors that affect the risk of high blood pressure [5–7]. Periodontal disease are common oral diseases with high prevalence worldwide [8]. Most studies show that there is a significant positive correlation between periodontal disease and hypertension [9–11]. Furthermore, there is a consistent association between cardiovascular disease (CVD) and oral health. One possible biological mechanism for this association is that the inflammatory mediators released into the local environment as a result of periodontitis could enter the blood stream and contribute to a systemic low-grade chronic inflammation known to be associated with an increased risk for future CVD [12]. This is mainly related to the low-grade systemic inflammation associated with poor periodontal health-characterized by increased C-reactive protein (CRP), interleukin-6 (IL-6), and fibrinogen levels [13].

It is well known that smoking is a risk for several diseases, including cardiovascular diseases [14] and oral diseases, especially periodontal diseases [15]. Epidemiological examinations reveal a strong correlation between smoking and inflammatory diseases of gingival and periodontal tissues and the mucosal surfaces [16]. Cigarette smoke has been shown to augment the production of pro-inflammatory cytokines such as tumor necrosis factor-α (TNF-α), interleukin (IL)-1, IL-6 and IL-8 and to decrease the levels of anti-inflammatory cytokines such as IL-10 [17]. Periodontitis is a chronic inflammatory disease, studies have shown that inflammation is the driving force behind neutrophil-mediated tissue degradation and alveolar bone loss in P. gingivalis-induced periodontitis [18]. Smoking increases the susceptibility of patients to infection of periodontopathogens and accelerates the progression of periodontitis via accelerating the destruction of periodontal supporting tissues, which increases the severity of periodontitis [19]. Pro-inflammatory T cell–derived cytokines such as interferon (IFN)-γ and TNF-α and IL-17 A exacerbate hypertensive responses mediating both endothelial dysfunction and cardiac injury [20]. The inflammatory response associated with periodontitis is also considered to be an important factor affecting the regulation of blood pressure [21].

At present, there are many researches on the influencing factors of hypertension at home and abroad, but there are few reports on the interaction between the influencing factors of hypertension. The risk factors of disease do not exist alone, but are the result of many factors working together. Due to the interaction of various factors, when multiple factors work together on a result, the influence of a single factor may be enhanced or diminished. Therefore, through the interaction between factors, the real relationship between factors and results can be reflected [22]. This study aimed to: (1) investigate the association between oral health and periodontal disease with the prevalence of hypertension; (2) explore the interaction effect of oral health, periodontal disease and smoking on hypertension based on the National Health and Nutrition Examination Survey (NHANES) database in the United States.

## Materials and methods

### Study population

NHANES data by the centers for disease control and prevention, national center for health statistics web site at https://www.cdc.gov/nchs/nhanes. It a regular survey conducted by the National Center for Health Statistics at the U.S. Centers for Disease Control and Prevention. For analysis, we used data from the 2009–2018 National Health and Nutrition Examination Survey (NHANES), a cross-sectional, nationally representative sample of the non-institutionalized U.S. adult population. During interviews, trained staff conducted questionnaires to collect self-reported information on social demographic and health-related behaviors and prescription drug use. During the clinical examination, trained technicians measured height, weight and blood pressure, and collected blood and urine samples for laboratory tests. All participants provided informed consent.

Because periodontal assessment was only performed in people over 30 years of age and older during this survey. We finally included 21,800 participants after excluded those without the blood pressure (n = 19,398), information for smoking, oral health, and periodontal disease (n = 1,090).

### Definition of variables

#### Blood pressure measurement

Blood pressure measurements were taken by trained personnel and/or physicians and all measurements were taken at mobile testing center. After resting in a sitting position for 5 min, blood pressure was measured with a mercury sphygmomanometer. A cuff of appropriate size was placed on the exposed right arm to obtain three consecutive blood pressure readings, and a fourth was performed if the blood pressure measurement was interrupted or incomplete. In our analysis, we calculated an average of these measurements for each participant. The reasons participants were thought to have high blood pressure were defined by any affirmative answer of the following questions: “Have you ever been told by a doctor or other health professional that you have hypertension, also called high blood pressure?”; “are you now taking prescribed medicine for high blood pressure?” ; or if they had a high biological measurement value (systolic blood pressure ≥ 140 mm Hg and/or diastolic blood pressure ≥ 90 mm Hg) [23].

#### Oral health and periodontal disease

In recent years, self-reported health status assessing systemic diseases and health-related conditions are widely used in populational investigations. Previous studies have shown that self-reported oral health questions and self-reported periodontal disease have good validity in larger populations [24–26]. Self-reported periodontal screening questions were adequately performed in identifying clinically periodontal disease [27]. Self-reported oral health is also an indicator with high reliability and validity, comparable to the results of an oral examination performed by a dentist [28, 29]. In conclusion, self-reported measures performed well in predicting oral health. However, the clinical measurement of periodontal disease only detects periodontitis as a single disease in NHANES study. The questionnaire defined periodontal disease as reflected by gum disease, including multiple gum diseases such as swollen gums, receding gums, sore or infected gums or loose teeth. Therefore, our study selected NHANES questionnaire data from 2009 to 2018 to reflect the relationship between oral health, periodontal disease and hypertension.

Oral health assessment uses the questionnaire survey results to conduct self-assessment through the following question: “Overall, how would you rate the health of your teeth and gums?” The answers were as follows: excellent, very good, good, fair and poor [30]. The oral health questionnaire was done before the physical examination, using the Computer-Assisted Personal Interviewing-CAPI (interviewer administered) system at home.

Periodontal disease assessment uses the questions: “Do you think you might have gum disease?” We treated this variable as a binary outcome: “self-aware of gum disease” (yes/no). If the answer to this question is yes, they are considered to have periodontal disease. Gum disease involves swollen gums, receding gums, sore or infected gums or loose teeth [31].

### Covariates

Potential confounding factors for adjustment were age (continuous), sex, race (mexican American, other hispanic, non-hispanic white, non-hispanic black and other race-including multi-racial), education (< high school, high school, > high school), body mass index (< 18.5, 18.5 ~ 24.9, 25 ~ 29.9, ≥ 30), alcohol consumption (no drinks, ≤ 1 drink/week, > 1 drink/week), smoking (yes/no), physical activity (active or inactive), sleep duration (< 7 h a night, 7 to 9 h a night, > 9 h a night), total cholesterol, HbA1c, creatinine, HDL, and triglycerides, sodium, fat, history of CVD (yes/no), diabetes (yes/no). Missing values for these covariates were treated as additional missing categories and their indicators dummy variables were included into the model.

### Statistical analysis

Participants were divided into two groups in accordance with their hypertension status: group with and without hypertension. We analyzed all the data using survey weights, strata, and primary sampling units created by NCHS to allow for national estimates according to NHANES analytic guidelines [32]. Means and standard errors (SE) were used to describe quantitative variables (age, etc.) and inter-group comparisons were performed using the T-test. The number (n) and percentage (%) of categorical data were calculated, and the comparison between groups was performed by Chi-square (χ2) test. Logistic regression models were used to assess the association between smoking, oral health, periodontal disease and the prevalence of hypertension. We conducted the sensitivity analysis on hypertension defined by measured blood pressure only (undiagnosed hypertension) and defined by physician diagnosed or treated disease. The effects of oral health and periodontal disease on hypertension under smoking status and age were analyzed by stratified analysis and interaction. We used SAS version 9.4 software (SAS Institute Inc, Cary, NC, USA) in all statistical analyses. Two-tailed *p*-values of < 0.05 were considered statistically significant.

## Results

Table 1 shows the general demographic characteristics of 21,800 participants. There are 48.40% male and 51.60% female. Of that, 11,017 (50.54%) were with hypertension and 10,783 (49.46%) without hypertension. Compered to persons without hypertension, persons in hypertension group tended to be older, smoke, have periodontal disease and fair/poor oral health, be more obese, have higher triglyceride, creatinine, HbA1c, and lower total-cholesterol and HDL.

**Table 1:** **Characteristics of overall participants according to hypertension status in NHANES, 2009–2018.** (N = 21,800)

Variables		Non-hypertension	Hypertension		*p-*value
No. of participants		10,783(49.46)	11,017(50.54)		
Age, y, mean (SE)		47.95(0.24)	59.04(0.21)		< 0.001
Sex, N (%)					0.015
Male		5140(46.74)	5412(48.93)		
Female		5643(53.26)	5606(51.07)		
Race/ethnicity, N(%)					< 0.001
Mexican American		1753(9.21)	1283(5.90)		
Other Hispanic		1193(6.14)	1060(4.90)		
Non-Hispanic White		4363(67.38)	4469(68.23)		
Non-Hispanic Black		1742(8.35)	2964(13.68)		
Other Race-Including Multi-Racial		1732(8.91)	1241(7.29)		
Smoking, N(%)					< 0.001
Yes		4520(42.01)	5398(49.76)		
No		6263(57.99)	5619(50.24)		
Periodontal disease, N(%)					< 0.001
Yes		1905(16.47)	2178(19.92)		
No		8878(83.53)	8839(80.08)		
Oral Health, N(%)					< 0.001
Excellent/Very good		3584(40.87)	3185(34.83)		
Good		3779(34.00)	3757(34.00)		
Fair		2374(17.53)	2620(19.91)		
Poor		1046(7.60)	1455(11.27)		
Education, N(%)					< 0.001
<High school		2374(13.77)	2923(17.13)		
High school		2147(19.45)	2669(25.11)		
>High school		6252(66.74)	5407(57.66)		
Missing		10(0.01)	18(0.11)		
BMI, N(%)					< 0.001
< 18.5		232(1.83)	312(2.41)		
18.5 ~ 24.9		3330(31.28)	1949(16.17)		
25 ~ 29.9		3757(35.15)	3450(31.41)		
≥ 30		3440(31.51)	5286(49.83)		
Missing		24(0.23)	20(0.18)		
Sleep duration, N(%)					< 0.001
< 7		7679(68.23)	7672(67.52)		
7 ~ 9		2737(28.93)	2747(27.53)		
> 9		367(2.84)	598(4.95)		
Alcohol consumption, N(%)					< 0.001
No drinks		337(3.07)	615(4.87)		
≤ 1 drink/week		4745(45.21)	4039(39.46)		
> 1 drink/week		2145(25.24)	2072(23.82)		
Missing		3556(26.48)	4291(31.84)		
Physical activity, N(%)					< 0.001
Inactive		4390(44.76)	3976(41.37)		
Active		6384(55.20)	7037(58.62)		
Missing		9(0.05)	4(0.01)		
Total cholesterol, mg/dl, mean (SE)		197.71(0.67)	195.03(0.80)		< 0.001
Triglyceride, mg/dl, mean (SE)		146.27(1.85)	172.62(1.90)		< 0.001
Creatinine, mg/dl, mean (SE)		0.85(0.00)	0.95(0.01)		< 0.001
HDL, mg/dl, mean (SE)		55.11(0.31)	52.64(0.29)		< 0.001
HbA1c, mg/dl, mean (SE)		5.55(0.01)	5.98(0.01)		< 0.001
Sodium, mg, mean (SE)		3575.03(25.86)	3476.48(25.77)		< 0.001
Fat, g, mean (SE)		85.55(0.66)	81.87(0.63)		< 0.001
History of CVD, N(%)					< 0.001
Yes		537(4.31)	2257(17.90)		
No		10,218(95.53)	8681(81.54)		
Missing		28(0.16)	79(0.55)		
Diabetes, N(%)					< 0.001
Yes		874(5.79)	2872(21.86)		
No		732(6.93)	1232(12.00)		
Missing		9177(87.28)	6913(66.14)		

All N are analytic sample N

SE, standard error of mean

All % and mean (SE) are weighted in order to represent the USA population

BMI, body mass index; HbA1c, glycohemoglobin A1c;

HDL, high-density lipoprotein

Table 2 shows the association between oral health, periodontal disease and the prevalence of hypertension. Compared with the excellent/very good of oral health, the multivariable-adjusted OR of good, fair, and poor were 1.13 (95% CI, 1.02–1.27), 1.30 (95% CI, 1.15–1.47), and 1.48 (95% CI, 1.22–1.79) (*p* for trend < 0.001) for hypertension, respectively. Compared without periodontal disease group, the multivariable-adjusted OR of periodontal disease for hypertension was 1.21 (95% CI ,1.09–1.35) (*p* for trend < 0.001). In the smoking group, compared with the excellent/very good of oral health, the multivariable-adjusted OR of good, fair, and poor were 1.13 (95% CI, 0.96–1.33), 1.11 (95% CI, 0.93–1.32), 1.31 (95% CI, 1.03–1.67) (*p* for trend = 0.013) for hypertension, respectively, and the multivariable-adjusted OR of periodontal disease for hypertension was 1.16 (95% CI, 0.98–1.37) (*p* for trend = 0.080), compared without periodontal disease. In the non-smoking group, compared with the excellent/very good of oral health, the multivariable-adjusted OR of good, fair, and poor were 1.12 (95% CI, 0.98–1.27), 1.49 (95% CI, 1.26–1.77), 1.62 (95% CI, 1.24–2.12) (*p* for trend = 0.032), and the multivariable-adjusted OR of periodontal disease for hypertension was 1.22 (95% CI, 1.01–1.47) (*p* for trend = 0.041), compared without periodontal disease group. The stratified analyses by the smoking shows the interactions between periodontal disease and smoking, oral health and smoking were *p* < 0.001. After subsequent adjustment for confounding’s, we found the effect of oral health and periodontal disease on hypertension was more significant among non-smoking persons.

**Table 2:** Multivariable odds ratios (ORs, 95%CI) of prevalence of hypertension and stratified by smoking status

	Cases/total participants	OR (95% CI)		
		Model 1	Model 2	Model 3
**Oral health**				
Excellent/ Very good	3185/6769	1.00	1.00	1.00
Good	3757/7536	**1.23(1.12–1.35)**	**1.14(1.03–1.26)**	**1.13(1.02–1.27)**
Fair	2620/4994	**1.48(1.32–1.65)**	**1.32(1.17–1.49)**	**1.30(1.15–1.47)**
Poor	1455/2501	**1.84(1.58–2.15)**	**1.49(1.24–1.79)**	**1.48(1.22–1.79)**
*P*-trend		**< 0.001**	**< 0.001**	**< 0.001**
**Non-Smoking**				
Excellent/ Very good	1801/4151	1.00	1.00	1.00
Good	2004/4320	**1.19(1.03–1.38)**	1.12(0.99–1.27)	1.12(0.98–1.27)
Fair	1293/2529	**1.20(1.02–1.42)**	**1.51(1.27–1.79)**	**1.49(1.26–1.77)**
Poor	521/882	**1.49(1.22–1.82)**	**1.67(1.29–2.16)**	**1.62(1.24–2.12)**
*P*-trend		**< 0.001**	**< 0.001**	**0.032**
**Smoking**				
Excellent/ Very good	1384/2618	1.00	1.00	1.00
Good	1753/3216	**1.22(1.07–1.38)**	1.12(0.96–1.31)	1.13(0.96–1.33)
Fair	1327/2465	**1.73(1.49–2.02)**	1.11(0.94–1.30)	1.11(0.93–1.32)
Poor	934/1619	**2.33(1.88–2.89)**	**1.29(1.02–1.63)**	**1.31(1.03–1.67)**
*P*-trend		**< 0.001**	0.03	**0.013**
*P*-interation	**< 0.001**			
**Periodontal disease**				
No	8839/17,717	1.00	1.00	1.00
Yes	2178/4083	**1.35(1.22–1.49)**	**1.20(1.08–1.34)**	**1.21(1.09–1.35)**
*P*-trend		**< 0.001**	**< 0.001**	**< 0.001**
**Non-Smoking**				
No	4664/10,018	1.00	1.00	1.00
Yes	955/1864	**1.41(1.20–1.66)**	1.19(0.99–1.44)	**1.22(1.01–1.47)**
*P*-trend		**< 0.001**	0.066	**0.041**
**Smoking**				
No	4175/7699	1.00	1.00	1.00
Yes	1223/2219	**1.24(1.06–1.44)**	1.14(0.98–1.34)	1.16(0.98–1.37)
*P*-trend		**0.006**	0.097	0.080
*P*-interation	**< 0.001**			

Model 1: adjusted for age, sex, race/ethnicity, education

Model 2: Model 1 plus BMI, sleep duration, alcohol intake, physical activity,

history of CVD, diabetes, sodium, fat

Model 3: Model 2 plus total cholesterol, creatinine, triglyceride, HbA1c, HDL.

We presented the stratified analyses on hypertension defined by measured blood pressure only and defined by physician diagnosed or treated disease in Supplemental table I and table II. We still found a positive association between oral health and undiagnosed hypertension or physician diagnosed and treated hypertension, but the periodontal disease is only associated with physician diagnosed and treated hypertension.

Table 3 shows multivariable odds ratios (ORs, 95%CI) of prevalence of hypertension according to oral health, periodontal disease status, and stratified by age. We found the interactions between oral health and age or between periodontal disease and age were *p* < 0.001. In the group younger than 45, compared with the excellent/very good of oral health, the multivariable-adjusted OR of good, fair, and poor were 1.11 (95% CI, 0.93–1.33), 1.27 (95% CI, 1.05–1.53), 1.71 (95% CI, 1.29–2.28) (*p* for trend < 0.001) for hypertension, respectively, and the multivariable-adjusted OR of periodontal disease for hypertension was 1.51 (95% CI, 1.27–1.79) (*p* for trend < 0.001), compared without periodontal disease. In the group older than 45, compared with the excellent/very good of oral health, the multivariable-adjusted OR of good, fair, and poor were 1.10 (95% CI, 0.98–1.24), 1.25 (95% CI, 1.07–1.46), 1.29 (95% CI, 1.07–1.55) (*p* for trend = 0.001), and the multivariable-adjusted OR of periodontal disease for hypertension was 1.05 (95% CI, 0.92–1.20) (*p* for trend = 0.444), compared without periodontal disease group. The interactions between periodontal disease and age, oral health and age were *p* < 0.001. After subsequent adjustment for confounding’s, we found the effect of oral health and periodontal disease on hypertension was more significant among the group younger than 45.

**Table 3:** Multivariable odds ratios (ORs, 95%CI) of prevalence of hypertension and stratified by age

	Cases/total participants	OR (95% CI)			
		Model 1	Model 2	Model 3	
**Oral health**					
**Age < 45**					
Excellent/ Very good	415/2053	1.00	1.00	1.00	
Good	552/2298	**1.23(1.04–1.46)**	1.12(0.94–1.34)	1.11(0.93–1.33)	
Fair	438/1598	**1.50(1.25–1.80)**	**1.32(1.11–1.58)**	**1.27(1.05–1.53)**	
Poor	218/640	**2.14(1.60–2.86)**	**1.81(1.35–2.41)**	**1.71(1.29–2.28)**	
*P*-trend		**< 0.001**	**< 0.001**	**< 0.001**	
**Age > = 45**					
Excellent/ Very good	2770/4716	1.00	1.00	1.00	
Good	3205/5238	**1.16(1.05–1.29)**	1.11(0.99–1.24)	1.10(0.98–1.24)	
Fair	2182/3396	**1.33(1.15–1.53)**	**1.26(1.08–1.45)**	**1.25(1.07–1.46)**	
Poor	1237/1861	**1.45(1.25–1.68)**	**1.31(1.11–1.54)**	**1.29(1.07–1.55)**	
*P*-trend		**< 0.001**	**< 0.001**	**0.001**	
*P*-interation	**<0.001**				
**Periodontal disease**					
**Age < 45**					
No	1243/5381	1.00	1.00	1.00	
Yes	380/1208	**1.71(1.43–2.04)**	**1.52(1.29–1.81)**	**1.51(1.27–1.79)**	
*P*-trend		**< 0.001**	**< 0.001**	**< 0.001**	
**Age > = 45**					
No	7596/12,336	1.00	1.00	1.00	
Yes	1798/2875	1.08(0.96–1.21)	1.05(0.93–1.18)	1.05(0.92–1.20)	
*P*-trend		0.21	0.444	0.444	
*P*-interation	**<0.001**				

Model 1: adjusted for sex, race/ethnicity, education

Model 2: Model 1 plus BMI, sleep duration, alcohol intake, physical activity, smoking

Model 3: Model 2 plus total cholesterol, creatinine, triglyceride, HbA1c, HDL.

## Discussion

In this study of nationally representative data from NHANES 2009 to 2018 on people over the age of 30 in the United States, we found a positive association between oral health and periodontal disease with hypertension. After subsequent adjustment for confounding’s, those with poor oral health or periodontal disease had a higher ORs for hypertension. And the effect of oral health and periodontal disease on hypertension was more significant among non-smoking persons and the group younger than 45. We also identified the interaction between periodontal disease and smoking, oral health and smoking, periodontal disease and age, oral health and age on hypertension.

Our result was consistent with the previous studies. In a large prospective cohort of 32,285 participants in France with a median follow-up of 8 years showed that arterial hypertension was associated with self-reported periodontal health, severe periodontitis (mPESS ≥ 5) at baseline was an independent predictor of arterial hypertension development, the multivariable HR (95% CI) was 1.84 (1.66–2.03) compared with mPESS less than 5 [33]. A 12-year longitudinal health-examinee cohort study in South Korea showed that periodontal disease was significantly positively related to hypertension compared with normal hypertension (OR = 1.04, 95% CI = 1.01–1.07, *p* < 0.014) after adjustment for sex, age, household income, insurance status, residence area, health status, and smoking status [34]. However, in another prospective cohort of 31,543 male professionals (dentists, pharmacists, optometrists, podiatrists, osteopaths, and veterinarians) aged 40–75 years in the United States, there was no significant relationship between self-reported periodontal disease at baseline and the occurrence of hypertension at 20 years of follow-up (RR = 1.04, 95% CI = 0.98–1.10) [35]. Unlike this study, our results showed a significant positive relationship between periodontal disease and hypertension. The differences with our study may be explained by the differences in baseline age (> 30 years old vs. 40–75 years old) and the study population (Americans over the age of 30 vs. male health professionals).

It has been indicated that smoking and age are important factors in the pathogeneses of hypertension [3, 6]. We stratified our analyses by the smoking status and age. Interestingly, the persons with poor oral health and periodontal disease in the non-smoking group and the group younger than 45 had a higher prevalence of hypertension in present study. This may be due to the following reasons: for smokers, it may be possible to follow dental advice longer after the diagnosis of poor oral health to increase oral health awareness, improve oral hygiene and smoking risk behaviors, which may lead smokers to subsequently reduce smoking or quit smoking and thus reduce the prevalence of hypertension; another explanation may be due to the statistical results only reflecting the large sample size (powers) or accidental findings. Participants in smoking group reported excellent oral health possibly because they were light smokers. However, some inevitable misclassification could have happened. But we still can observe a real association between oral health and hypertension among non-smokers. A cross-sectional study from Taiwan on Cardiorespiratory Fitness and Hospitalization Events in Armed Forces showed that systolic and diastolic blood pressure in 1,123 military participants were not associated with the risk of stage II/III periodontitis [36], which was contrary to our study. The differences may be explained by the differences in the study population (military VS general population) and definition of periodontitis (clinical measurement VS self-reported). The effects of smoking on oral health range from life threatening conditions like oral cancer to tooth staining with potential jeopardies to oral health morbidity and tooth mortality. Various studies have shown smoking as a risk factor leading to an increase in the incidence and progression of periodontitis, contributing to tooth loss and edentulism as compared to non-smokers [37–39]. Therefore, reducing or not smoking will further improve oral health and reduce the prevalence of hypertension [40].

Our findings also in line with the previous study which is showed the association between dental calculus and hypertension (HTN) in Taiwanese military personnel aged 19–45 years. The study showed that poor oral health manifested by dental calculus was associated with a greater likelihood of CHTN (combined HTN) in young adults, and that there was a dose-dependent relationship between the number of teeth with any dental calculus and total HTN and CHTN [41]. A few studies revealed that the presence of dental calculus represents poor oral hygiene and is related to the greater risk of HTN [7]. In other words, the worse the oral health, the higher the prevalence of hypertension.

The biologic mechanisms for oral health or periodontal disease and hypertension are not well understood. Some studies have found that patients with primary arterial hypertension in poor oral status are more susceptible to oxidative stress, which may result in increased levels of reactive metabolites of oxygen, lipid peroxidation, total antioxidant capacity. This could result in the inactivation of prostacyclin and NO. Hence, an enhancement of peripheral vascular resistance and hypertension [42, 43]. Periodontitis is a chronic infection that causes inflammation, particularly, the inflammatory response accompanying periodontitis has been proposed as an important factor that may exert adverse effects on the regulation of BP [21]. It is reported that periodontitis attenuates endothelium - dependent vasodilation in rats [44, 45].The causes were increased systemic inflammatory biomarkers (CRP and IL-6), increased production of vascular superoxide free radicals, worsening lipid status, and decreased expression of vascular nitric oxide synthase-3 (NOS-3) [45]. However, periodontitis may also lead to an increased systemic immune response in patients [44]. A cross-sectional study found a positive correlation between gingival fluid TNF-α levels and blood pressure (*p* < 0.05), inducing a double inflammatory effect in patients with both periodontitis and hypertension [46]. Thus, inflammatory mediators such as C-reactive protein (CRP), tumor necrosis factor -α (TNF-α) and interleukin-6 (IL-6) may be the link between periodontitis and hypertension, and smoking may promote the co-occurrence and development of the two diseases as a common risk factor.

Dentists have been reported to be effective in motivating patients to quit smoking, with brief, repeated motivational interventions by physicians and other health professionals (including dentists) achieve long-term cessation success rates of about 16–20% [47]. A cross-sectional study using a theoretical model to study the importance of periodontal status on oral health-related quality of life in 195 adults with systemic arterial hypertension. The results showed that poor oral health-related quality of life was directly predicted by smoking (β = 0.172), more lost teeth (β = 0.273), more decayed teeth (β = 0.198) and worse periodontal status (β = 0.293) [48]. A cohort study of 8,139 participants with a median follow-up time of 6.59 years, 1,215 incident hypertension cases were identified. After adjustment for potential confounders, they found that brushing teeth once a day was associated with a 23% reduction in risk of hypertension (HR: 0.77, 95% CI: 0.60–0.98, *p* < 0.05), while brushing teeth at least twice a day was associated with a 45% reduction (HR: 0.55, 95% CI: 0.42–0.73, *p* < 0.001) [49]. Therefore, we can reduce the prevalence of hypertension by quitting smoking and taking regular oral hygiene care.

The strengths of our study are found in the following aspects. First, we used five cycles of high-quality, representative data on Americans from NHANES, which was large and multi-stage [50]. Secondly, previous studies have shown that periodontal disease and oral health are risk factors for hypertension, but no studies have shown that there is an interaction between them and smoking on hypertension. Here, we report for the first time an interactive effect of oral health and smoking, periodontal disease and smoking on the prevalence of hypertension in individuals over 30 years in the United States. Finally, self-rated oral health has the advantage of embodying the perspective of the participant and is considered to be a valid, reliable, and inexpensive way to measure overall oral health [51].

We declare several potential limitations in our study. Firstly, the oral health assessment in this study, the measure of periodontal disease was self-reported, it was subjective rather than objective, and there were individual differences [30, 31]. Self-reported measures may underestimate or overestimate the association between oral health/periodontal disease and hypertension. Secondly, this was a cross-sectional survey with poor accuracy and universality, so we cannot determine causality or exclude a two-way relationship. Finally, the study only included people over 30 years old, 18–29 people were not included in the analysis, and the results could not cover this population. Now the behavior of smoking is gradually becoming younger, because NHANES periodontal examination is conducted in people over 30 years of age and older, so this problem is inevitable in this study.

## Conclusion

We found an association between oral health and periodontal disease and the prevalence of hypertension. There exists interactive effect of periodontal disease and smoking, oral health and smoking, periodontal disease and age, oral health and age on hypertension in American population over 30 years of age and older, and the effect of oral health and periodontal disease on hypertension was more significant among non-smoking persons and the group younger than 45.

## Electronic supplementary material

Below is the link to the electronic supplementary material.

Supplementary Material 1

## Data Availability

The datasets presented in this study can be found in online repositories. The names of the repository/repositories and accession number(s) can be found at: https://wwwn.cdc.gov/nchs/nhanes.

## Abbreviations

BP: Blood pressure
CRP: C-reactive protein
CVD: Cardiovascular disease
HbA1c: Glycosylated Hemoglobin
HDL: High density lipoprotein
HR: Hazard ratio
IFN: Interferon
IL-6: Interleukin-6
IL: Interleukin
mPESS: Severe periodontitis
N: Number
NCHS: National Center for Health Statistics
NHANES: National Health and Nutrition Examination Survey
NOS-3: Nitric oxide synthase-3
OR: Odds ratio
SE: Means and standard errors
TNF-α: Factor-α
U.S.: United States
