# Supplementary material for: The association of periodontal disease and oral health with hypertension, NHANES 2009–2018
Source: BMC Public Health. 2023 Jun 12;23:1122. doi: 10.1186/s12889-023-16012-z (PMC10262359; doi:10.1186/s12889-023-16012-z)
Supplement: Supplementary file 1 — Supplementary Material 1 [file 12889_2023_16012_MOESM1_ESM.pdf]

Supplemental table I. Multivariable odds ratios (ORs, 95%CI) of prevalence of undiagnosed hypertension.

	Cases/total participants	OR (95% CI)		
		Model 1	Model 2	Model 3
**Oral health**				
Excellent/ Very good	499/4083	1.00	1.00	1.00
Good	553/4332	1.14(0.95-1.38)	1.15(0.95-1.39)	1.13(0.92-1.39)
Fair	382/2756	**1.34(1.09-1.65)**	**1.33(1.08-1.64)**	**1.30(1.05-1.61)**
Poor	194/1240	**1.44(1.06-1.96)**	**1.39(1.03-1.88)**	**1.42(1.04-1.93)**
P-trend		**<0.001**	**0.002**	**0.003**
**Periodontal disease**				
No	1344/10222	1.00	1.00	1.00
Yes	284/2189	0.89(0.74-1.08)	0.88(0.73-1.05)	0.88(0.73-1.06)
P-trend		0.222	0.135	0.159

Model 1: adjusted for age, sex, race/ethnicity, education.
Model 2: Model 1 plus BMI, sleep duration, alcohol intake, physical activity.
Model 3: Model 2 plus total cholesterol, creatinine, triglyceride, HbA1c, HDL.

Supplemental table II. Multivariable odds ratios (ORs, 95%CI) of prevalence of physician diagnosed and treated hypertension.

	Cases/total participants	OR (95% CI)		
		Model 1	Model 2	Model 3
**Oral health**				
Excellent/ Very good	2686/6270	1.00	1.00	1.00
Good	3204/6983	**1.25(1.12-1.38)**	**1.19(1.07-1.32)**	**1.17(1.04-1.31)**
Fair	2238/4612	**1.50(1.33-1.70)**	**1.43(1.27-1.64)**	**1.39(1.21-1.59)**
Poor	1261/2307	**1.93(1.64-2.26)**	**1.81(1.52-2.15)**	**1.66(1.38-1.99)**
P-trend		**<0.001**	**<0.001**	**<0.001**
**Periodontal disease**				
No	7495/16373	1.00	1.00	1.00
Yes	1894/3799	**1.44(1.31-1.59)**	**1.42(1.28-1.56)**	**1.37(1.24-1.53)**
P-trend		**<0.001**	**<0.001**	**<0.001**

Model 1: adjusted for age, sex, race/ethnicity, education.
Model 2: Model 1 plus BMI, sleep duration, alcohol intake, physical activity.
Model 3: Model 2 plus total cholesterol, creatinine, triglyceride, HbA1c, HDL.
